# Supplementary material for: Insights from a global snapshot of the change in elective colorectal practice due to the COVID-19 pandemic
Source: PLoS One. 2020 Oct 8;15(10):e0240397. doi: 10.1371/journal.pone.0240397 (PMC7544024; doi:10.1371/journal.pone.0240397)
Supplement: S1 File — (PDF) [file pone.0240397.s001.pdf]

PanSurg ColoQ - An international cross-sectional survey of colorectal cancer management during the COVID-19 pandemic

Dear colleague,

We are facing significant challenges in the management of colorectal cancer during the COVID-19 pandemic. As such, PanSurg.org is conducting an international survey of surgeons managing patients witl non-metastatic colorectal cancers, documenting their views, resource availability and current treatment strategies in the context of the pandemic. We aim to inform on current practice to better understand the impact of the COVID-19 pandemic on patients with colorectal cancer. All data will be open source and available at PanSurg.org

Data will be available in realtime for the surgical community to interact with: https:/
https://www.pansurg.org/ColoQ_data

This online survey will be distributed via social media to international bodies with relevant members who treat colorectal cancers, such as ESCP, ACPGBI and others.

This survey should take 5 minutes to complete.

In a few weeks we will ask you to complete a follow-up survey so we can compare the results as the situation develops. We would be grateful if you could provide an email contact. Please note your responses will be strictly anonymous.

Many thanks for taking the time to complete this survey at such a challenging time.

Yours sincerely,

James Kinross on behalf of the PanSurg Collaborative

https://pansurg.org
* Required

1. By checking this box, I certify that I am a Consultant or Attending Surgeon who currently treats colorectal cancers and I give my consent freely to participate in this *

Check all that apply.

☐ Yes

2. Please provide an email address so we can contact you strictly for the purpose of a follow-survey in a few weeks? *

Baseline Information

3. Age

4. Gender

Mark only one oval.

- Male
- Female
- Prefer not to say

5. Country *

Mark only one oval.

- Australia
- Austria
- Belgium
- Canada
- China
- Denmark
- Finland
- France
- Germany
- Greece
- Hungary

- India
- Ireland
- Italy
- Japan
- Netherlands
- New Zealand
- Norway
- Pakistan
- Portugal
- Romania
- Russia
- Singapore
- South Africa
- South Korea
- Spain
- Sweden
- Switzerland
- Turkey
- United Kingdom
- United States of America
- Other: ______________________

6. Speciality *

Mark only one oval.

- Colorectal Surgeon
- General Surgeon
- Other: ______________________

7. Year completed Specialist Surgical Training

8. Hospital Type *

Mark only one oval.

- Academic / tertiary / teaching
- Local / district / community

9. Total number of hospital beds *

Mark only one oval.

- 0-99
- 100-200
- 201-500
- 501-1000
- >1000

10. Total number of critical care beds *

Mark only one oval.

- 0-9
- 10-20
- 21-50
- 51-100
- >100

11. Do you have patients infected with COVID-19 currently being treated at your hospital? *

Mark only one oval.

- Yes
- No
- Unknown

12. How many patients infected with COVID-19 are currently being treated at your hospital? *

Mark only one oval.

- 0-9
- 10-20
- 21-50
- 51-100
- 101-200
- >200
- Unknown

13. Using the following system, what is the current CRITCON level of your hospital? *

COVID-19 Pandemic CRITCON Levels

Please declare CRITCON level and for CRITCON 1, 2 or 3 the staffing level A or B

DEFINITION	STATUS
**Normal – *'Business as usual'***	
• Normal, able to meet all critical care needs, without impact on other services<br>• Normal winter levels of non-clinical transfer and other overflow activity.	CRITCON 0
**Low Surge – *'Bad winter'***	
• Usual funded critical care capacity full. Some non-clinical transfers<br>• Phased reduction of elective work to support critical care, by local decision.	CRITCON 1
**Medium Surge – *'Unprecedented'***	
• Usual funded critical care capacity full – overflow into quasi-critical care areas (theatre recovery, other acute care areas). High level of non-clinical transfers<br>• Trusts beginning mutual aid<br>• Elective surgery & medical procedures minimised to urgent/cancer & lifesaving only.	CRITCON 2
**High Surge – *'Full stretch'***	
• Expansion into non-critical care areas (e.g. wards) and/or use of paediatric facilities for adult critical care. Trust operating at or near maximum physical capacity.<br>• Maximum mutual aid between Trusts, with network and regional NHSE co-ordination.<br>• The prime imperative in CRITCON 3 is to prevent any single trust entering CRITCON 4	CRITCON 3
**Triage – *'Last resort'***	
• Resources overwhelmed. Possibility of triage by resource (non-clinical refusal or withdrawal of critical care due to resource limitation).<br>• This must **only** be implemented on national directive from NHSE and in accordance with national guidance.	CRITCON 4
**Staff Declaration: CRITCON 1,2 & 3 SHOULD BE FURTHER CATEGORISED A OR B**	
• Adhering to BACCN / ICS staffing recommendations or unit norm	A
• Staffing below BACCN / ICS staffing recommendations or unit norm	B

Mark only one oval.

- CRITCON 1A
- CRITCON 1B
- CRITCON 2A
- CRITCON 2B
- CRITCON 3A
- CRITCON 3B
- CRITCON 4

Diagnostic and Staging Modalities for CRC

14. Are MDT / tumour boards at your centre running as usual? *

Mark only one oval.

- Yes
- No - limited numbers in the room but face-to-face meeting
- No - virtual meeting (video or telephone linkage)
- No - they have been stopped

15. Does outpatient diagnostic endoscopy remain available as usual? *

Mark only one oval.

- Yes
- No - limited or delayed availability
- No - unavailable during COVID-19 pandemic

16. Is CT scanning available as usual? *

Mark only one oval.

- Yes
- No - limited or delayed availability
- No - unavailable during COVID-19 pandemic

17. Is rectal MRI available as usual? *

Mark only one oval.

- Yes
- No - limited or delayed availability
- No - unavailable during COVID-19

18. Is PET scanning available as usual? *

Mark only one oval.

- Yes
- No - limited or delayed availability
- No - unavailable during COVID-19 pandemic

Therapeutic Strategies for CRC

19. Prior to COVID-19 pandemic, was therapeutic endoscopy (i.e EMR / ESD / stenting) routin[illegible] available? *

Mark only one oval.

- Yes
- No

20. During COVID-19 pandemic: is therapeutic endoscopy (i.e EMR / ESD / stenting) routinely available? *

Mark only one oval.

- Yes
- Yes but limited compared to normal limited
- No

21. To deal with the COVID-19 pandemic are you adapting the use of neoadjuvant therapies f curable rectal cancer? *

Mark only one oval.

- Yes - decreased use of neoadjuvant therapies
- Yes - increased use of neoadjuvant therapies
- No
- Other: ____________________

22. To deal with the COVID-19 pandemic are you adapting the use of neoadjuvant therapies f curable colonic cancer? *

Mark only one oval.

- Yes - decreased use of neoadjuvant therapies
- Yes - increased use of neoadjuvant therapies
- No
- Other: ____________________

23. Are you using colonic stenting more frequently during the pandemic as a strategy to dela curative surgery for colon cancer? *

Mark only one oval.

- Yes - using colonic stenting more frequently
- No - no change in use of colonic stents

24. What additional delay is there between neoadjuvant therapy and surgical intervention du to COVID-19? *

Mark only one oval.

- No delay
- <2 weeks
- 2-4 weeks
- 4-8 weeks
- 8-12 weeks
- >12 weeks

Surgical Management of CRCs

25. How are cancer resections being screened for COVID-19 preoperatively? *

Mark only one oval.

- 1 COVID swab (PCR)
- > 1 COVID swab (PCR)
- CT chest
- COVID swabs (PCR) and CT chest
- Antibody testing
- No screening
- Other: ____________________

26. There have been reports of nosocomial COVID-19 infection on the postoperative ward increasing mortality of surgical patients. Is this currently a consideration at your institutio[illegible] when deciding to perform elective colorectal resection? *

Mark only one oval.

- Yes
- No
- Other: ______________________

27. Do you discuss the potential risk of nosocomial COVID-19 infection on the postoperative ward with patients preoperatively? *

Mark only one oval.

- Yes
- No
- Other: ______________________

28. Have you moved your elective resectional work to a "clean" site *

Mark only one oval.

- Yes
- No

29. Due to limitations during the COVID-19 pandemic, has it been necessary to prioritise som patients awaiting curative colorectal resection ahead of others awaiting curative resectio *

Mark only one oval.

- Yes - based on local guidance
- Yes - based on national guidance
- No
- Other: ____________________

30. What criteria should be used to prioritise patients awaiting curative resection? *

Mark only one oval per row.

	1st	2nd	3rd	4th	5th	6th
Age						
Fitness and Co-morbidities						
Stage of Disease						
Expected difficulty of case						
Extended delay since initial diagnosis or neoadjuvant therapy						
Need for an ICU bed						

31. Who is generally performing surgical resections during the COVID-19 crisis? *

Mark only one oval.

- Consultants assisted by consultants (dual operating)
- Consultants assisted by registrars/fellows
- Trainees under consultant supervision
- N/a - resectional surgery has stopped

32. Have concerns about COVID-19 aerosolisation influenced the proportion of resections performed laparoscopically? *

Mark only one oval.

- Yes - laparoscopy has ceased entirely
- Yes - laparoscopy reduced
- Yes - laparoscopy increased
- No change

33. Is filtered air extraction being used during laparoscopy? *

Mark only one oval.

- Yes
- No
- N/a - laparoscopy has stopped

34. Have limitations during the COVID-19 pandemic caused you to reconsider which patients are sent to critical care (HDU/ICU) postoperatively? *

Mark only one oval.

- No
- Yes - all patients receive ward-based care postoperatively
- Yes - higher threshold must be met for postoperative critical care
- Other: ____________________

35. How have stoma formation rates changed since COVID-19 pandemic? *

Check all that apply.

- [ ] More likely to defunction left-sided anastamoses
- [ ] More likely to perform Hartmann's procedure rather than perform left-sided anastomosis
- [ ] Decrease in stoma use
- Other: [ ] ____________________

Thank you

Thank you for completing the survey during this challenging time

This content is neither created nor endorsed by Google.

Google Forms
